# Differential Roles of Glycogen Synthase Kinase 3 Subtypes Alpha and Beta in Cortical Development

**DOI:** 10.3389/fnmol.2017.00391

**Published:** 2017-11-28

**Authors:** Yan-xia Ma, Xiu-li Wang, Jian-quan Chen, Bin Li, Eun-Mi Hur, Saijilafu

**Affiliations:** ^1^Department of Orthopaedics, The First Affiliated Hospital, Orthopaedic Institute, Soochow University, Suzhou, China; ^2^Center for Neuroscience, Brain Science Institute, Korea Institute of Science and Technology, Seoul, South Korea; ^3^Convergence Research Center for Diagnosis, Treatment and Care System of Dementia, Korea Institute of Science and Technology, Seoul, South Korea; ^4^Department of Neuroscience, Korea University of Science and Technology, Daejeon, South Korea

**Keywords:** GSK3α, GSK3β, cortical development, neurogenesis, β-catenin

## Abstract

Glycogen synthase kinases 3 (GSK3) α and β are expressed in the nervous system, and disruption of GSK3 signaling has been implicated in a wide range of neurodevelopmental and psychiatric disorders. Although several studies have established a role of GSK3 signaling in the nervous system, much less is known about isoform-specific functions. Here, we have examined the role of GSK3α and GSK3β in the developing neocortex by performing *in utero* electroporation with specific small interfering RNAs targeting each isoform. We found that depletion of either GSK3α or GSK3β commonly promoted the proliferation of neural progenitor cells in the ventricular zone, but at later stages, knocking down of each isoform resulted in distinct outcomes. In particular, the transformation of radial progenitors to intermediate progenitor cells was promoted in GSK3α-depleted cells, but markedly prevented in GSK3β-depleted cells. Moreover, knocking down of GSK3β but not GSK3α prevented the generation of upper-layer Cux1^+^ neurons. Consistent with the distinct outcomes, protein levels of c-Myc and β-catenin, well-known substrates of GSK3, were differentially affected by depletion of GSK3α and GSK3β. Together, these results suggest that GSK3α and GSK3β might play distinct roles in the genesis and differentiation of neuronal lineage cells during neocortex development by differential regulation of downstream signaling pathways.

## Introduction

Neural progenitor cells (NPCs) are self-renewing and multipotent cells that proliferate, migrate, and differentiate in a defined temporal sequence, thereby generating layer-specific classes of excitatory neurons in the cerebral cortex (Temple, 2001; Guillemot et al., 2006; Guillemot, 2007). Among the progenitors are radial glial cells (RGCs), which reside in the ventricular zone (VZ). RGCs undergo stereotypical patterns of symmetrical and asymmetrical cell divisions in the developing brain and give rise to diverse types of neurons, while maintaining a pool of progenitors that can self-renew. RGCs can also generate neurons indirectly via intermediate progenitor cells (IPCs), which usually undergo one symmetric terminal division in the VZ and the subventricular zone (SVZ) (Kowalczyk et al., 2009), producing two neurons that migrate to the cortical plate (CP) (Haubensak et al., 2004; Noctor et al., 2004; Englund et al., 2005; Kowalczyk et al., 2009). Abnormalities in any of such processes can result in dysfunctions of the brain and lead to neurological diseases, including a wide range of neurodevelopmental and psychiatric disorders (Raedler et al., 1998; Reif et al., 2006; Manzini and Walsh, 2011; Rossi et al., 2011; Eisch and Petrik, 2012).

Glycogen synthase kinases 3 (GSK3) α and β are serine/threonine protein kinases that play a crucial role in multiple signaling pathways, including Wnt/β-catenin, Notch, receptor tyrosine kinase, G-protein-coupled receptor, and Sonic hedgehog (Doble and Woodgett, 2003; Kim and Snider, 2011; Valvezan and Klein, 2011; McCubrey et al., 2016). In the brain, both GSK3α and GSK3β are expressed (Yao et al., 2002), and disruption of GSK3 signaling has been implicated in a number of neurological diseases, such as schizophrenia (Emamian et al., 2004; Mao et al., 2009), major depression (Li and Jope, 2010), bipolar disorder (Klein and Melton, 1996; Chenn and Walsh, 2002; Valvezan and Klein, 2011), and neurodegenerative diseases (Hooper et al., 2008; Lee et al., 2016). To date, several studies have clearly established a role of GSK3 signaling in the nervous system (Yokota et al., 2009; Hur and Zhou, 2010; Fang et al., 2013; Morgansmith et al., 2014; Jung et al., 2016). It has been shown that knocking out both GSK3α and GSK3β in target cells during development (*Gsk3a^-/-^*; *Gsk3b^loxP/loxP^*; *nestin-Cre* or *Gsk3a^-/-^*; *Gsk3b^loxP/lox^*; *Neurod6-Cre*) results in massive disorganization of the brain structure. Knocking out both GSK3s in the progenitor cells (*Gsk3a^-/-^*; *Gsk3b^loxP/loxP^*; *nestin-Cre*) at early developmental stages causes substantial hyperproliferation of progenitor cells (Kim et al., 2009), and deleting both genes in new-born neurons (*Gsk3a^-/-^Gsk3b^loxP/loxP^:Neurod6-Cre*) at later stages interferes with proper migration and dendritic arborization of excitatory neurons (Morgansmith et al., 2014). In contrast to the severe structural defects induced by double knockout, no major developmental malformations in the brain have been reported when either of the genes is deleted in NPCs (Kim et al., 2009), which might be due to molecular compensation. However, to the best of our knowledge, this has never been formally tested, and it remains unknown if GSK3α and GSK3β play redundant or distinct roles in the developing nervous system.

In the present study, we have analyzed isoform-specific roles of GSK3α and GSK3β in the developing neocortex by acutely depleting the target isoform via *in utero* electroporation, which has been suggested to circumvent the possible compensatory effects of general gene-knockout approaches. To our surprise, we found that although depletion of either GSK3α or GSK3β caused similar effects on the proliferation of NPCs, knocking down of each isoform caused distinct outcomes in the genesis of IPCs. Knocking down GSK3β but not GSK3α specifically prevented the conversion of radial progenitors to IPCs and further differentiation into upper-layer cortical neurons. Moreover, depletion of GSK3α and GSK3β differentially regulated the protein levels of c-Myc and β-catenin, well-known substrates of GSK3. These findings provide evidence that GSK3α and GSK3β play overlapping but distinct roles in neocortex development.

## Materials and Methods

### Antibodies

The following primary antibodies were used in this study: rabbit GSK3α and GSK3β antibodies (1:200 CST; #4337; #9315), rat anti-5-bromo-2-deoxyuridine (BrdU) antibody (1:100; Covance; MMS-139S), rabbit anti-Tbr2 (T-brain gene-2) antibody (1:100; Abcam; ab23345); rabbit anti-Cux1 antibody (1:80 Santa Cruz Biotechnology; sc-13024), rabbit anti-c-Myc antibody (1:200; GeneTex GTX103436), rabbit anti-β-catenin antibody (CST; #8480), and rabbit anti-GAPDH antibody (1:1000; Abcam; ab181603). The secondary antibodies conjugated with Alexa fluorophores 488 or 568 were directed against the IgGs of the primary antibody species (1:500; Invitrogen).

### Small Interfering RNA

The small interfering RNAs (siRNAs) against GSK3α and GSK3β (ON-TARGET plus SMART POOL) were from Thermo Scientific Dharmacon (Chicago, IL, United States). The sequences of GSK3α siRNA duplexes were: 5′-GUA CUA CCG UGC UCC AGA ATT-3′ (forward); 5′-UUC UGG AGC ACG GUA GUA CTT-3′ (reverse); 5′-CGU GAC AGC GGG AAG GUG A TT-3′ (forward); 5′-UCA CCU UCC CGC UGU CAC GTT-3′ (reverse); 5′-GAU UAC ACC UCG UCC AUC GTT-3′ (forward); 5′-CGA UGG ACG AGG UGU AAU CTT-3′ (reverse); 5′-GUG GUC GGC UGG CUG UGU ATT-3′ (forward); 5′-UAC ACA GCC AGC CGA CCA CTT-3′ (reverse); GSK3β siRNA duplexes were: 5′-GGA CCC AAA UGU CAA CUA TT-3′ (forward); 5′-UAG UUU GAC AUU UGG GUC CTT-3′ (reverse); 5′-CCA CAG GAA GUC AGU UAU ATT-3′ (forward); 5′-UAU AAC UGA CUU CCU GUG GTT-3′ (reverse); 5′-UCA GAA GUC UAG CCU AUA UTT-3′ (forward); 5′-AUA UAG GCU AGA CUU CUG ATT-3′ (reverse); 5′-GAU UAC ACG UCC AGU AUA GTT-3′ (forward); 5′-CUA UAC UGG ACG UGU AAU CTT-3′ (reverse). The sequences of scrambled control siRNA were: 5′-UUC UCC GAA CGU GUC AGG UTT-3′ (forward) and 5′-AGG UGA CAC GUU CGG AGA ATT-3′ (reverse).

### Ethics Statement

The mice used in this study were ICR mice. All mice were handled and treated according to the animal care and handling protocols approved by the Institutional Animal Care and Use Committee of Soochow University. In all experiments, the pregnant mice were anesthetized with a mixer of isoflurane (11.5%) and oxygen isoflurane (30%) or 3.6% chloral hydrate.

### Immunohistochemistry

Embryonic brains were fixed in 4% paraformaldehyde at 4°C for 24 h, followed by dehydration in 20 and 30% sucrose solution (w/v), each for 24 h. Brains were cryosectioned in 14 μm thickness, and the brain sections were washed three times in PBS containing 0.3% Triton X-100, followed by blocking for 1 h in PBS containing 10% fetal bovine serum (FBS). Sections were incubated with the indicated primary antibodies overnight at 4°C, washed in PBS containing 0.3% Triton X-100 and then incubated with the corresponding secondary antibodies for 1 h. Sections were stained with Hoechst (Beyotime, C1022) for 20 min at room temperature. The sections were mounted with mounting medium (Vector Labs, H-1400) after washing in PBS containing 0.3% Triton X-100. For BrdU staining, the sections were treated with 2 N HCl for 20 min in a 60°C thermostat drier before blocking with PBS containing 10% FBS.

### *In Utero* Electroporation

*In utero* electroporation procedure was performed as described (Saito and Nakatsuji, 2001). We used pEX-4 plasmid containing a reporter gene EGFP downstream of CMV promoter in all experiments to visualize transfected cells (C05004, GenePharma Company, China). Embryonic day 14.5 (E14.5) pregnant mice were anesthetized with a mixer of isoflurane (11.5%) and oxygen isoflurane (30%) or 3.6% chloral hydrate. The abdomen was cleaned with 75% ethanol and disinfected with a LIONSER^®^ compound iodine cotton swab. A 3-cm midline laparotomy was performed, and the uterus was taken out. Microinjection was carried out with PCR micropipettes (Drummond^®^; #5-000-1001 × 10). Lateral ventricles of E14.5 embryos were injected with a mixture of 1 μl of pEX-4 (2.5 μg/μl) and 1 μl of siRNA (100 μM). siGSK3α/β and scrambled control siRNA were injected into different sides of lateral ventricles. Electric pulses (voltage: 45 V; pulse length: 50 ms; number: 5; interval: 950 ms) were applied by an ECM 830 Electroporator (BTX, Holliston, MA, United States). The electroporated pregnant mice were placed on a soft bedding with a warming blanket (37°C) until the dams were fully awake. The electroporated mice were sacrificed and analyzed at E18.5. Electroporated mice were from the same litter.

### Cell Line and Transfection

Cath.-a-differentiated (CAD) cells are a variant of a CNS catecholaminergic cell line that expresses pan-neuronal markers and differentiates to extend processes in serum-free conditions (Qi et al., 1997). CAD cells were cultured in DMEM/F-12 medium supplemented with 10% FBS, 2 mM L-glutamine, and 100 U/ml penicillin and streptomycin (all from Invitrogen). Cells were cultured at 37°C in a CO_2_-humidified incubator. Lipofectamine 2000 reagent was used for siRNA transfection according to the instructions provided by the manufacturer.

### Primary Culture of Cortical Neurons

Primary cortical neurons dissected from E15 mice as previously reported (Jang et al., 2016) with minor modifications. Cerebral cortices of E15 mice were dissected out and digested with trypsin (Gibco; 12604-021) for 5 min at 37°C. Enzyme-digested cortices were washed three times with MEM and dissociated in MEM. The dissociated neurons were centrifuged to remove the supernatant and resuspended in electroporation buffer containing siRNAs against GSK3 or control siRNA. Electroporation was performed immediately using an ECM 830 Electroporator (BTX, Holliston, MA, United States) with electrical pulses: five 50 ms pulses at 112 V with 950 ms interval. After electroporation, cells were immediately mixed with a suitable volume of prewarmed Neurobasal Medium supplemented with GlutaMAX and B27 and the neurons were plated on polylysine-coated plastic dishes. At 4–6 h after electroporation, the medium was changed to remove the electroporation buffer, and then the cells were cultured for 3 days.

### Western Blot Analysis

For western blot analysis, cells were lysed in RIPA buffer. Protein concentration was determined by using the BCA Kit (Beyotime; P0012). Proteins were separated in a 10% SDS-PAGE gel, and transferred to a PVDF membrane, which was blocked (1 h) with 5% non-fat milk in TBST with 0.05% Tween 20. The membranes were sequentially incubated with the indicated primary antibodies in TBST containing 5% non-fat milk overnight at 4°C, followed by incubation with horseradish peroxidase-conjugated secondary antibodies for 2 h at room temperature. ECL Western Blotting Detection Reagents (Millipore; WBKLS0500) were used to visualize immunoreactive proteins. The band intensity was analyzed by Image J.

### BrdU Labeling and Proliferation Analysis

For BrdU labeling experiments, either pEX-4 plus siGSK3α/β or pEX-4 plus control siRNA was transfected into E14.5 embryos by *in utero* electroporation. After 4 days (at E18.5), pregnant mice were injected intraperitoneally with BrdU (50 mg/kg body weight) and 2 h later, animals were sacrificed, and embryos were prepared for immunohistochemical analysis as described above. Cell proliferation was calculated as the percentage of BrdU^+^ GFP^+^ double positive cells among total cells positive for GFP.

### Image Acquisition and Analysis

Fluorescent images were acquired using an AxioImager M1 epifluorescence microscope (Carl Zeiss). Images were taken with a 10× or a 20× objective. All images were acquired at a 1388 × 1040 pixel resolution. When analyzing fluorescent intensity, fluorescent exposure settings were kept the same, and all images were processed in parallel. Images were analyzed using AxioVision Rel.4.7 software (Carl Zeiss) and image J. To increase clarity, images were processed using Adobe Photoshop CS5 software and processing was applied equally across the entire image.

### Quantification and Statistical Analysis

Data were collected from three independent experiments (for western blots) or three pairs of electroporated brain littermates (*N* = 3, for immunostaining). For each brain, eight slices (*n* = 8) were analyzed. For quantification of cells in the brain sections obtained from mice electroporated with either pEX-4 plus siGSK3α/β or pEX-4 plus control siRNA, the number of cells co-expressing a maker of interest (BrdU, Tbr2, or Cux1) together with GFP was counted in the dorsal cortex of telencephalon and presented as a percentage value of total GFP^+^ cells. All data were collected from the three independent experiments. Two-tailed Student’s *t*-test or one-way ANOVA was performed using SPSS software followed by Bonferroni’s post-test as *post hoc* test for comparing multiple groups. Statistics was applied to *N* and values are presented as mean ± SEM, and significance was set at *p* < 0.05 (^∗^*p* < 0.05; ^∗∗^*p* < 0.01; ^∗∗∗^*p* < 0.001).

## Results

### Validation of siRNAs against GSK3α and GSK3β

We first examined where GSK3 isoforms were expressed in the developing mouse neocortex. We found that GSK3α and GSK3β were highly expressed in NPCs in the germinal zone and IPCs in the SVZ of the dorsal telencephalon. GSK3α and GSK3β-immunoreactive fluorescence signals were detected in the apical wall of the VZ of the dorsal telencephalon, but no immunoreactive fluorescence signals were observed when the coronal sections of the dorsal telencephalon were stained with non-immune control immunoglobulin (data not shown).

**FIGURE 1:**
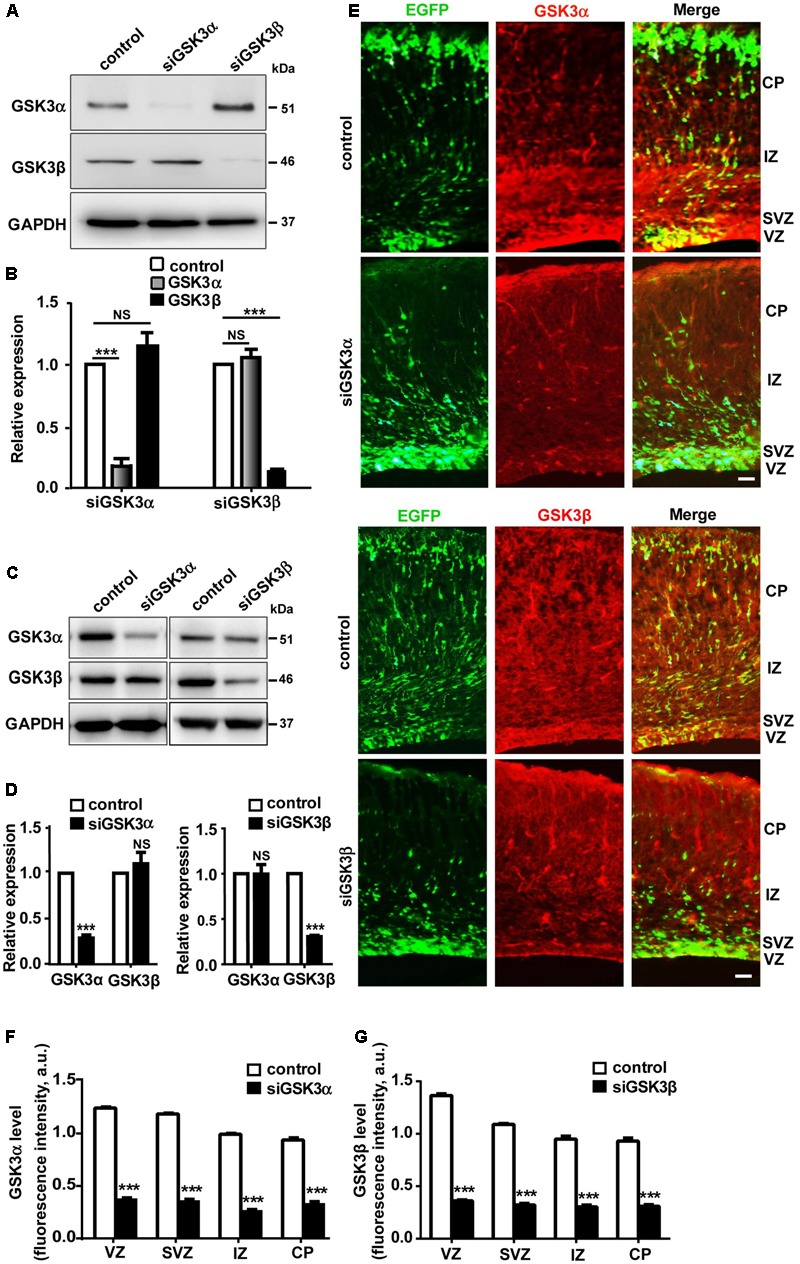
Validation of siRNAs targeting either GSK3α or GSK3β. **(A,B)** Western blot analysis of CAD cells transfected with siRNAs against GSK3α or GSK3β, or scrambled siRNA as a control. Shown are representative images **(A)** and quantification of the blots **(B)** from three independent experiments. **(C,D)** Western blot analysis of cortical neurons transfected with siRNAs against GSK3α or GSK3β, or scrambled siRNA as a control. Shown are representative images **(C)** and quantification of the blots **(D)** from three independent experiments. **(E–G)** E14.5 embryos were electroporated *in utero* with pEX-4 together with siRNAs against either GSK3α or GSK3β or with control siRNAs, and mice were sacrificed at E18.5 to examine the effect of GSK3 depletion on neocortical development. Brain sections were immunostained with anti-GSK3α or anti-GSK3β antibodies, as indicated. Representative images **(E)** and quantification of fluorescence intensity of GSK3α **(F)** or GSK3β **(G)** signals after immunostaining are shown. VZ, ventricular zone; SVZ, subventricular zone; IZ, intermediate zone; CP, cortical plate. Scale bar, 20 μm. ^∗^*p* < 0.05; ^∗∗^*p* < 0.01; ^∗∗∗^*p* < 0.001; NS, non-significant; one-way ANOVA **(B)**, Student’s *t*-test **(D,F,G)**.

To explore the possible role of GSK3α and GSK3β in cerebral cortex development, we used siRNAs targeting either GSK3α (siGSK3α) or GSK3β (siGSK3β). In a catecholaminergic cell line CAD, where transfection efficiency reaches ∼80% with lipid-based transfection (Byun et al., 2012), we confirmed that siGSK3α specifically and effectively (79 ± 3.6%) knocked down the target isoform without affecting GSK3β, and likewise, siGSK3β downregulated GSK3β (81 ± 2.5%) without altering the expression of GSK3α (**Figures 1A,B** and Supplementary Figure S1). We also confirmed the efficacy and specificity of siGSK3α or siGSK3β in primary cortical neurons (**Figures 1C,D** and Supplementary Figure S1). In primary cortical neurons, knocking down efficacies were lower than those achieved in CAD cells, probably because of a lower transfection efficiency. Notably, in both CAD cells and primary cortical neurons, the extent of knockdown achieved by siGSK3α and siGSK3β toward the target isoform were similar, providing a basis for comparing the effect of the two siRNAs. Using GSK3 siRNAs, we performed *in utero* electroporation at E14.5 together with GFP and confirmed that substantial knockdown of the target GSK3 isoform was achieved in the developing cortex (**Figures 1E–G** and Supplementary Figure S2).

### GSK3α and GSK3β Regulate Neuronal Migration in the Developing Cerebral Cortex

**FIGURE 2:**
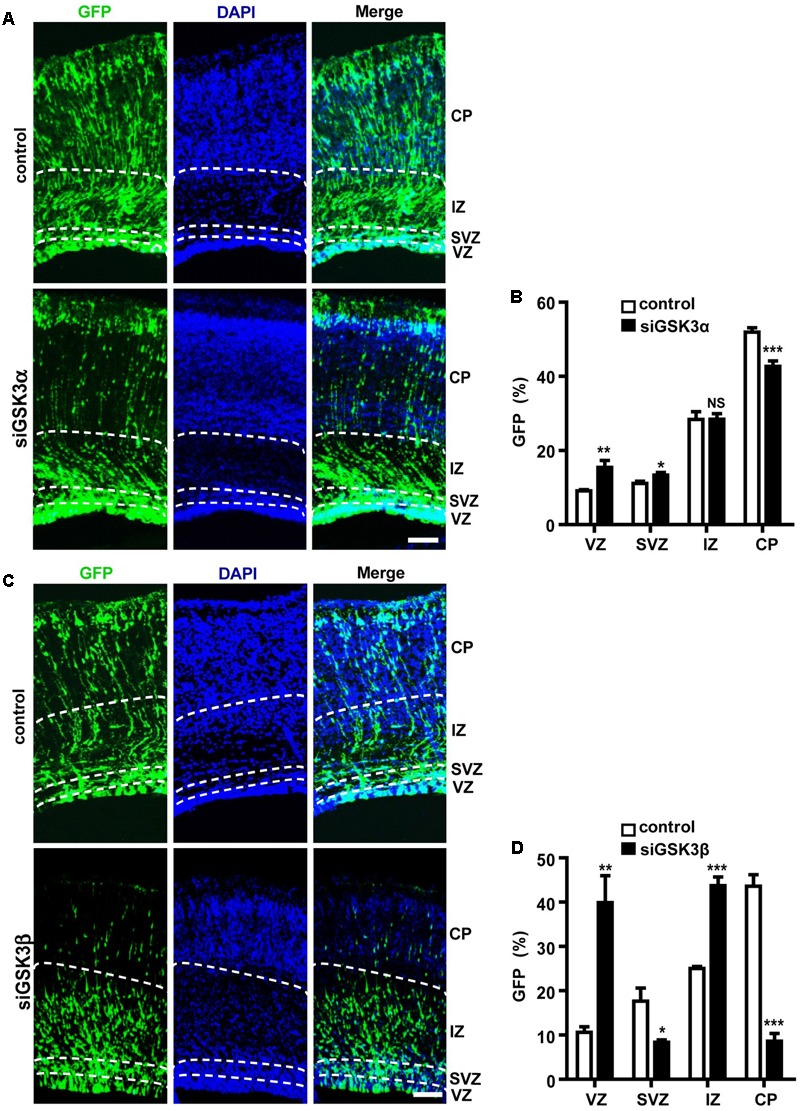
Both GSK3α and GSK3β are essential for proper migration. **(A–D)** E14.5 embryos were electroporated *in utero* with pEX-4 together with siRNAs against either GSK3α **(A,B)** or GSK3β **(C,D)** or with control siRNAs, and mice were sacrificed at E18.5. Coronal sections of the dorsal telencephalic cortex were stained with DAPI. **(B,D)** Quantification of the percentage of GFP^+^ cells in each layer. VZ, ventricular zone; SVZ, subventricular zone; IZ, intermediate zone; CP, cortical plate. Scale bars, 100 μm. Results are mean ± SEM. *N* = 3 brains, *n* = 8 slices from each brain. ^∗^*p* < 0.05; ^∗∗^*p* < 0.01; ^∗∗∗^*p* < 0.001; NS, non-significant, one-way ANOVA.

In the E14.5 embryonic cortex, most NPCs in the VZ divide asymmetrically to generate another NPC and either a postmitotic neuron or an IPC, which will then divide again to generate two postmitotic neurons in the SVZ (Haubensak et al., 2004; Noctor et al., 2004). IPCs populate the SVZ, and postmitotic neurons journey through the intermediate zone (IZ) toward the developing CP (Molyneaux et al., 2007). When we electroporated E14.5 embryos with pEX-4 and examined the locations of GFP^+^ cells at E18.5, GFP-expressing cells were present throughout the cortical layers but localized mostly to the IZ and the CP. When GSK3α was depleted by *in utero* electroporation at E14.5, more cells remained in the VZ and the SVZ, and fewer cells migrated into the CP by E18.5 as compared to control brains that had been transfected with scrambled, control siRNAs (**Figures 2A,B** and Supplementary Figure S3). We found a much severe migration defect in siGSK3β-transfected cells. When GSK3β was depleted, the percentage of GFP^+^ cells remaining in the VZ was four times higher than that of control brains, and few GFP^+^ cells reached the CP (**Figures 2C,D** and Supplementary Figure S3). These results show that depletion of GSK3α or GSK3β traps cells in the VZ and prevents cell migration toward the CP layer.

### Depletion of GSK3α and GSK3β Enhances Proliferation of Progenitors

**FIGURE 3:**
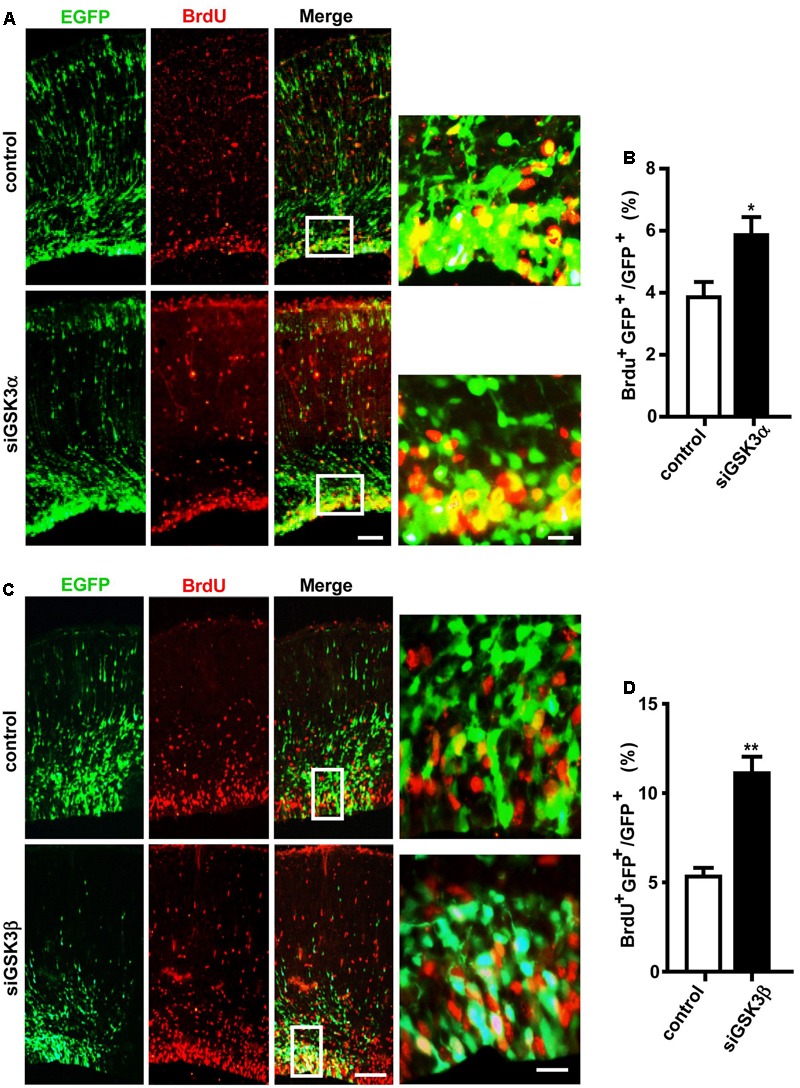
Knockdown of GSK3α or GSK3β promotes progenitor proliferation. E14.5 embryos were electroporated *in utero* with pEX-4 together with siRNAs against either GSK3α **(A,B)** or GSK3β **(C,D)**, or with control siRNAs. At E18.5, pregnant mice were injected intraperitoneally with BrdU, animals were sacrificed 2 h later and then coronal sections were stained for BrdU. Representative images **(A,C)** and quantification of BrdU^+^ cells **(B,D)** are shown. The number of BrdU^+^ GFP^+^ cells was counted and presented as a percentage of GFP^+^ cells. Scale bars, 100 μm (left) and 20 μm (right, inset enlarged at left). Results are mean ± SEM. *N* = 3 brains, *n* = 8 slices from each brain. ^∗^*p* < 0.05; ^∗∗^*p* < 0.01, Student’s *t*-test.

GFP^+^ cells remaining in the VZ and the SVZ after GSK3 depletion likely correspond to cells that maintain a progenitor identity capable of self-renewal and reentering the cell cycle. To test this, we performed *in utero* electroporation at E14.5 with pEX-4 together with siGSK3α, siGSK3β, or control siRNAs, and then at 4 days after electroporation, we labeled the embryos with BrdU. BrdU incorporates into cells that are in S-phase of the cell cycle and thus marks actively proliferating cells. Two hours after injection of BrdU into the pregnant dam, embryos were dissected, and brain sections were subjected to immunostaining with BrdU antibodies. We detected a higher percentage of GFP^+^ cells co-labeled with BrdU in the dorsal cortex of the mice electroporated with either siGSK3α or siGSK3β, as compared to control siRNAs (**Figures 3A–D** and Supplementary Figure S4). These results indicate that more cells were undergoing division after depletion of GSK3α or GSK3β, suggesting that both GSK3α and GSK3β inhibit the proliferation of progenitors in the developing cortex.

### GSK3 Isoforms Play Opposing Roles in the Transformation of Radial Progenitors to INPs

**FIGURE 4:**
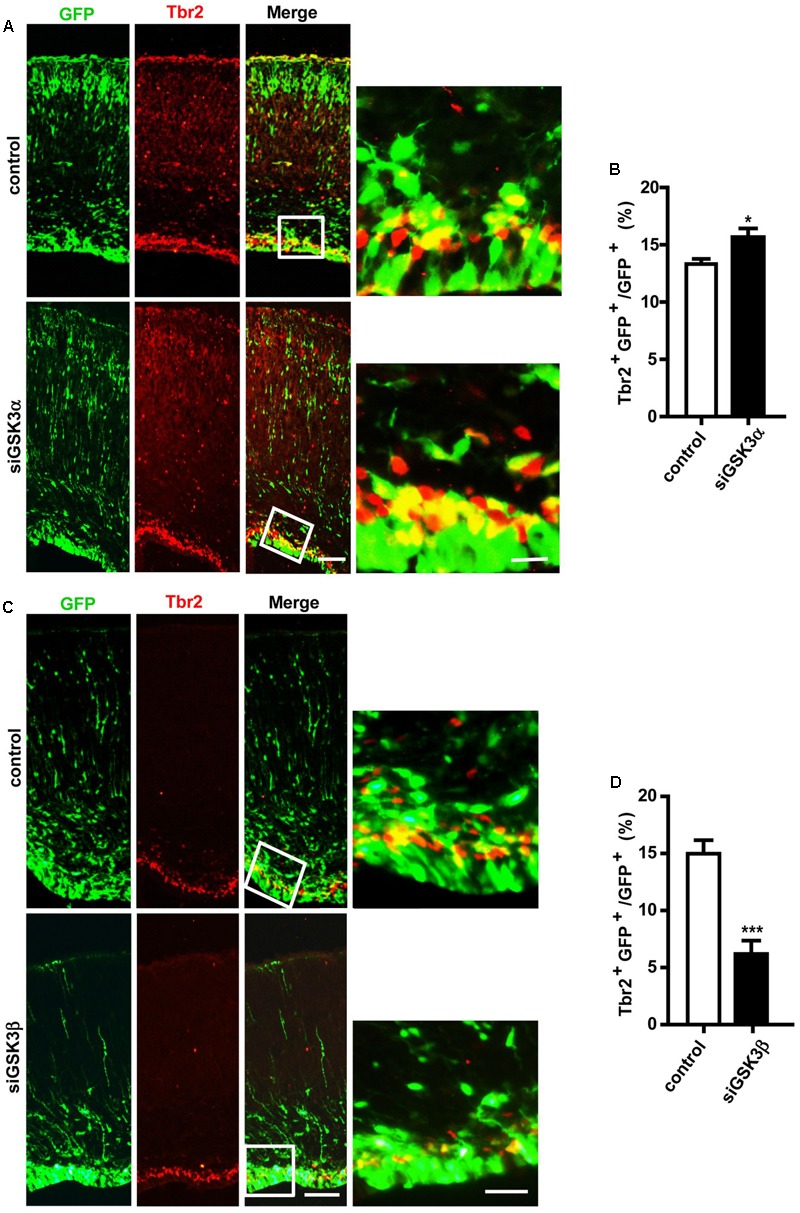
GSK3α and GSK3β play opposing roles in the genesis of intermediate neuronal progenitors. **(A–D)** E14.5 embryos were electroporated *in utero* with pEX-4 together with siRNAs against either GSK3α **(A,B)** or GSK3β **(C,D)** or with control siRNAs, and mice were sacrificed at E18.5. Coronal sections of the dorsal telencephalic cortex were stained for Tbr2. Representative images **(A,C)** and quantification of Tbr2^+^ cells **(B,D)** are shown. The number of Tbr2^+^ GFP^+^ cells was counted and presented as a percentage of GFP^+^ cells. Scale bars, 100 μm (left) and 20 μm (right, inset enlarged at left). Results are mean ± SEM. *N* = 3 brains, *n* = 8 slices from each brain. ^∗^*p* < 0.05; ^∗∗∗^*p* < 0.001, Student’s *t*-test.

Our findings show that depletion of GSK3α or GSK3β enhances the proliferation of progenitors (**Figures 3A–D**). Cortical neurons are generated from IPCs in the SVZ, which are derived from RGCs in the VZ or directly from RGCs (Guillemot et al., 2006). To examine the effect of GSK3 depletion on the genesis of IPCs, we performed *in utero* electroporation at E14.5 with pEX-4 and siGSK3α, siGSK3β or control siRNAs, and then at E18.5 brain sections were stained for Tbr2, a marker of IPCs. The percentage of Tbr2 and GFP double-positive cells were increased in the siGSK3α-transfected brains, as compared to control brains that had been electroporated with pEX-4 and scrambled siRNA (**Figures 4A,B** and Supplementary Figure S5). By contrast, brains transfected with pEX-4 and siGSK3β showed a substantial decrease in the percentage of Tbr2^+^ GFP^+^ cells, as compared to control brains (**Figures 4C,D** and Supplementary Figure S5). Although depletion of GSK3α and GSK3β commonly promoted the proliferation of progenitors in the VZ, knocking down of the two isoforms caused different outcomes in terms of Tbr2 staining. These results show that the conversion of radial progenitors to INPs is enhanced by knocking down GSK3α but suppressed by GSK3β depletion, suggesting distinct roles of the GSK3 isoforms in the developing cortex.

### Depletion of GSK3α and GSK3β Has Distinct Effects on the Generation of Upper-Layer Cortical Neurons

**FIGURE 5:**
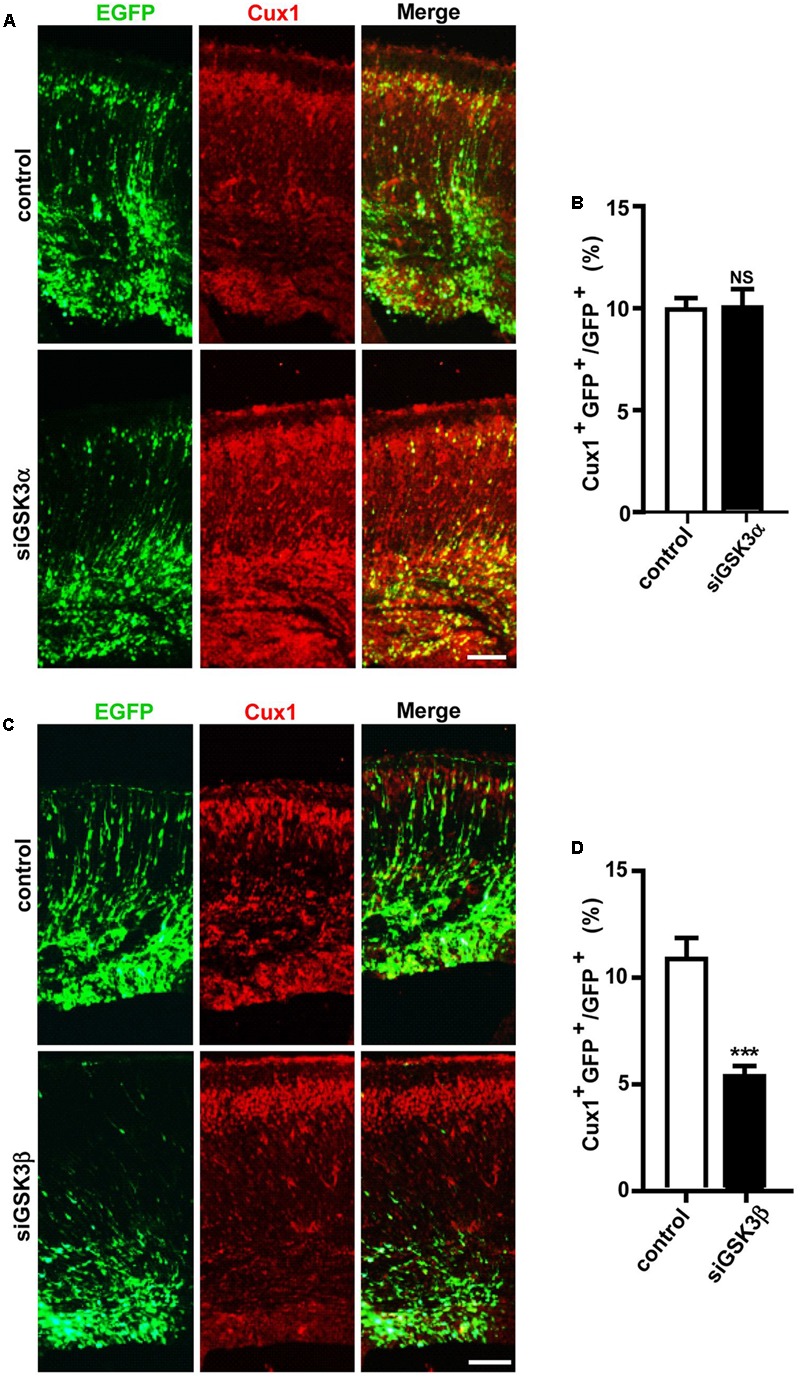
GSK3β depletion inhibits the generation of upper-layer cortical neurons. **(A–D)** E14.5 embryos were electroporated *in utero* with pEX-4 together with siRNAs against either GSK3α **(A,B)** or GSK3β **(C,D)** or with control siRNAs, and mice were sacrificed at E18.5. Coronal sections of the dorsal telencephalic cortex were stained for Cux1. Representative images **(A,C)** and quantification of the Cux1^+^ cells **(B,D)** are shown. The number of Cux1^+^ GFP^+^ cells was counted and presented as a percentage of GFP^+^ cells. Scale bars, 100 μm. Results are mean ± SEM. *N* = 3 brains, *n* = 8 slices from each brain. ^∗∗∗^*p* < 0.001. NS, non-significant, Student’s *t*-test.

IPCs in the SVZ migrate to the upper layers of the cerebral cortex and undergo differentiation during development (Kwan et al., 2012). We thus next examined how depletion of the GSK3 isoforms affected further migration and differentiation steps. When we immunostained the brain sections for Cux1, which is expressed in excitatory neurons that populate layers II–IV of the neocortex, we found that the percentages of Cux1^+^ GFP^+^ cells were markedly reduced by siGSK3β but unaffected by siGSK3α (**Figures 5A–D** and Supplementary Figure S6). These results again show distinct outcomes caused by knocking down of either GSK3α or GSK3β in the developing cortex.

### Effects of Knocking Down Both GSK3α and GSK3β on Cortical Development

**FIGURE 6:**
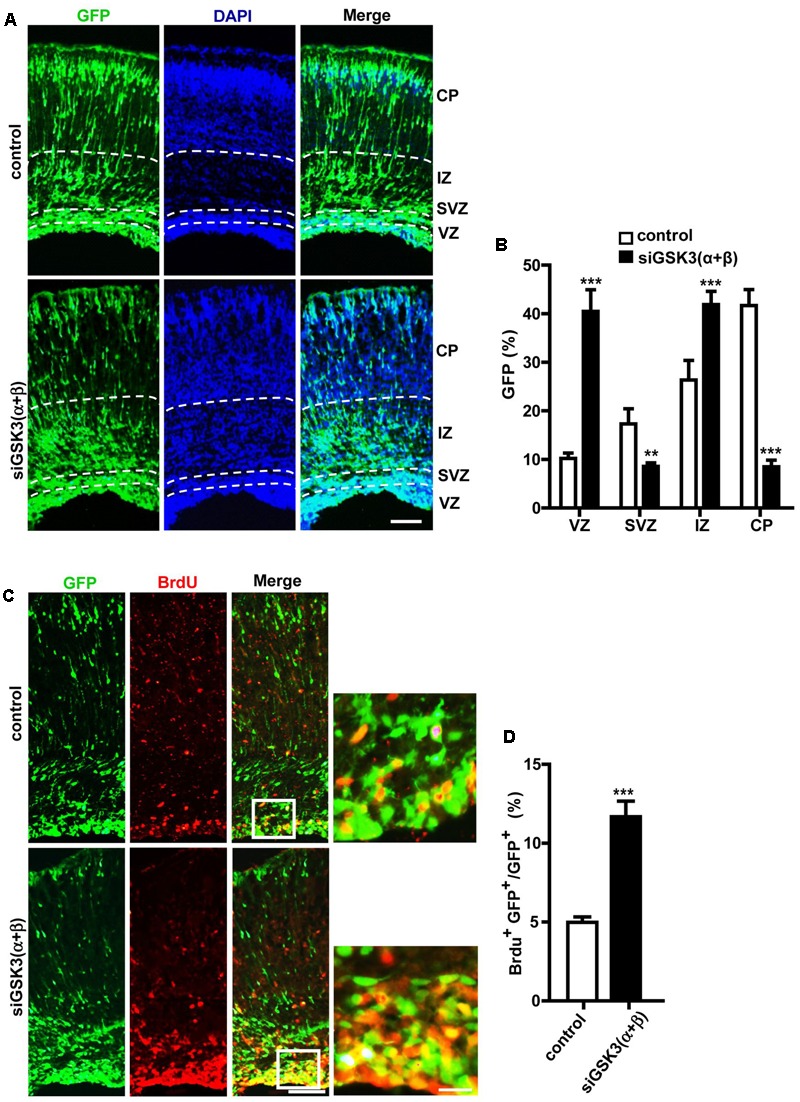
Effects of depleting both GSK3α and GSK3β on migration and proliferation. **(A–D)** E14.5 embryos were electroporated *in utero* with pEX-4 together with siRNAs against both GSK3α and GSK3β or with control siRNAs, and mice were sacrificed at E18.5. Coronal sections of the dorsal telencephalic cortex were collected and stained with DAPI. Representative images **(A)** and quantification of the percentage of GFP^+^ cells in each layer **(B)** are shown. VZ, ventricular zone; SVZ, subventricular zone; IZ, intermediate zone; CP, cortical plate. **(C,D)** For BrdU labeling, pregnant mice were injected intraperitoneally with BrdU at E18.5, and animals were sacrificed 2 h later. Representative images **(C)** and quantification of BrdU^+^ cells **(D)** are shown. The number of BrdU^+^ GFP^+^ cells was counted and presented as a percentage of GFP^+^ cells. Scale bars, 100 μm (left) and 20 μm (right, inset enlarged at left). Results are mean ± SEM. *N* = 3 brains, *n* = 8 slices from each brain. ^∗∗^*p* < 0.01; ^∗∗∗^*p* < 0.001; one-way ANOVA **(B)**, Student’s *t*-test **(D)**.

**FIGURE 7:**
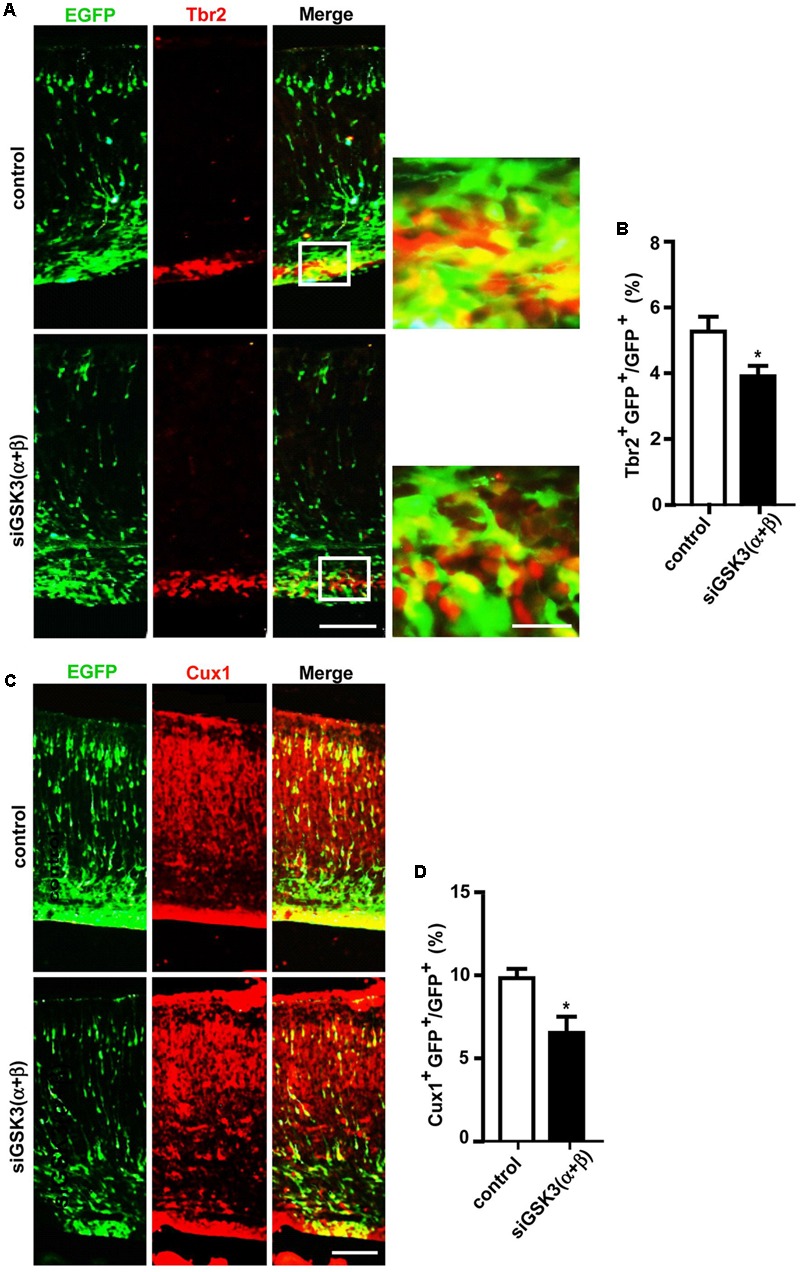
Depletion of both GSK3α and GSK3β prevents generation of IPCs and further differentiation. **(A–D)** E14.5 embryos were electroporated *in utero* with pEX-4 together with siRNAs against both GSK3α and GSK3β or with control siRNAs, and mice were sacrificed at E18.5. Coronal sections of the dorsal telencephalic cortex were stained for Tbr2 **(A,B)** or Cux1 **(C,D)**. Representative images **(A,C)** and quantification of Tbr2^+^ **(B)** or Cux1^+^ **(D)** cells are shown. Scale bars, 100 μm (left) and 20 μm (right, inset enlarged at left). Results are mean ± SEM. *N* = 3 brains, *n* = 8 slices from each brain. ^∗^*p* < 0.05, Student’s *t*-test.

Next, we examined how knocking down both isoforms together affected cerebral cortex development. For this purpose, E14.5 embryos were electroporated *in utero* with a mixture containing pEX-4 and both of the siRNAs (siGSK3α and siGSK3β) or pEX-4 and scrambled siRNA as a control. When brain sections were prepared from E18.5 embryos, we detected substantially higher percentages of GFP^+^ cells in the VZ and the IZ but fewer cells in the SVZ and the CP, as compared to control group (**Figures 6A,B** and Supplementary Figure S7). Knocking down of both siGSK3α and siGSK3β together markedly increased the percentage of BrdU^+^ cells (**Figures 6C,D** and Supplementary Figure S7) but decreased Tbr2^+^ (**Figures 7A,B** and Supplementary Figure S8) or Cux1^+^ cells (**Figures 7C,D** and Supplementary Figure S8), suggesting that GSK3 activity was required for the conversion of radial progenitor cells into IPCs and upper-layer cortical neurons. These results were consistent with a previous study from double knockout mice (*Gsk3a^-/-^*; *Gsk3b^loxP/loxP^*; *nestin-cre*), which specifically deleted *Gsk3b* in the progenitor cells in a *Gsk3a* null background (Kim et al., 2009). Notably, the changes in the distribution of GFP^+^ cells, as well as the effects on BrdU^+^, Tbr2^+^, and Cux1^+^ cells induced by depletion of both GSK3α and GSK3β were recapitulated by knocking down GSK3β alone (see **Figures 2D, 3D, 4D, 5D**). Given that siGSK3α and siGSK3β downregulated the target gene to similar extents (see **Figure 1**), these results suggest that isoform GSK3β might play a dominant role in the regulation of cell migration and fate during cortical development.

### c-Myc and β-Catenin Are Differentially Regulated by GSK3α and GSK3β

**FIGURE 8:**
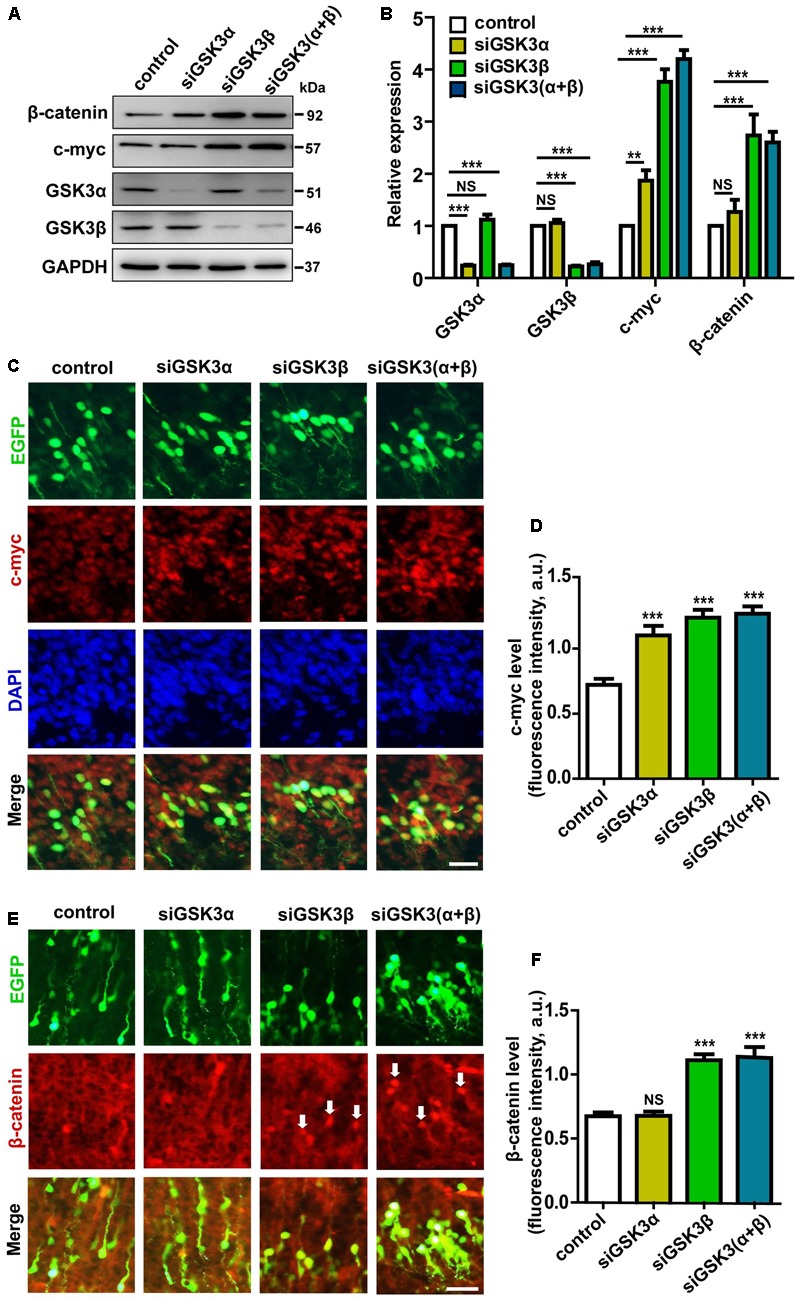
Knocking down GSK3α and GSK3β controls the level of c-Myc and β-catenin. **(A,B)** Cortical neurons were transfected with siRNAs against GSK3α, GSK3β, or both, or with scrambled siRNAs, as a control. Neurons were then harvested at 48 h after transfection and subjected to western blot analysis using antibodies against GSK3α, GSK3β, c-Myc, β-catenin, and β-actin as a loading control. Shown are representative images **(A)** and quantification of the blots **(B)** from three independent experiments. **(C–F)** E14.5 embryos were electroporated *in utero* with pEX-4 together with siRNAs against both GSK3α and GSK3β or with control siRNAs, and mice were sacrificed at E18.5. Coronal sections of the dorsal telencephalic cortex were stained for c-Myc **(C)** or β-catenin **(E)**. Quantification of the fluorescence intensity of c-Myc **(D)** or β-catenin **(F)** in cortical plate is shown. Scale bar, 20 μm. ^∗^*p* < 0.05; ^∗∗^*p* < 0.01; ^∗∗∗^*p* < 0.001; NS, non-significant; one-way ANOVA with Bonferroni.

Myc transcription factors, including c-Myc, N-Myc, and L-Myc, have been studied extensively as regulators of cell cycle progression and proliferation. In the developing nervous system, c-Myc and N-Myc are expressed, especially in proliferating cells, and Myc transcription factors have been shown to control the capacity of neural stem cells to self-renew and differentiate (Nagao et al., 2009). Notably, it is well documented that GSK3β phosphorylates and regulates the stability of c-Myc and N-Myc (Gregory et al., 2003; Sjostrom et al., 2005). Therefore, Myc proteins are obvious candidates to mediate GSK3 regulation of migration. In primary cortical neurons, we found that knocking down either GSK3α or GSK3β augmented the protein level of c-Myc (**Figures 8A,B** and Supplementary Figure S1). Interestingly, siGSK3β produced a much prominent enhancement, and the extent of increase induced by depletion of both GSK3α and GSK3β together was comparable to that caused by siGSK3β alone. In the developing cortex, we observed similar effects after *in utero* electroporation of siGSK3α, siGSK3β, or both, showing increased c-Myc protein level and a stronger elevation induced by siGSK3β (**Figures 8C,D** and Supplementary Figure S9) These results are consistent with the migration data in that a much severe migration defect was induced by siGSK3β as compared to siGSK3α (see **Figure 2**) and that the effect of knocking down both GSK3α and GSK3β could be recapitulated by depletion of GSK3β alone (see **Figures 2, 6B**).

Wnt/β-catenin signaling is also known to control migration of neural progenitors and β-catenin is a well-established substrate of GSK3 (Woodhead et al., 2006). We thus examined the effects of siGSK3α and siGSK3β on the level of β-catenin. Differential effects of knocking down GSK3α and GSK3β was much more evident in that only siGSK3β but not siGSK3α led to a significant elevation in the protein level of β-catenin both in cell culture (**Figures 8A,B** and Supplementary Figure S1) and the developing cortex (**Figures 8E,F** and Supplementary Figure S9). Together, these findings provide further support for the distinct roles of GSK3α and GSK3β and suggest that the differential effects of knocking down GSK3α or GSK3β on cell migration and fate in the developing cortex might have resulted from differential activation of the downstream signaling pathways.

## Discussion

The genes that encode GSK3α and GSK3β are commonly referred to as isoforms, but they are actually paralogs derived from duplication of an ancestral gene. GSK3α and GSK3β share 98% homology in their kinase domains but only 36% identity in their N- and C-termini (Woodgett, 1990), and the major difference between the two is a glycine-rich extension at the N-terminal that is present only in GSK3α. The facts that *Gsk3a^-/-^* mice are viable (Macaulay et al., 2007) but *Gsk3b^-/-^* mice die at late embryonic stages (Hoeflich et al., 2000) suggest that the two isoforms are not interchangeable. However, surprisingly little is known about differential regulation or isoform-specific roles of GSK3α and GSK3β.

Behavioral analyses of *Gsk3a* knockout mice and *Gsk3b* heterozygote knockout mice suggest that GSK3α and GSK3β might play distinct roles in the brain (O’Brien et al., 2004; Bersudsky et al., 2008; Kaidanovich-Beilin et al., 2009). Because *Gsk3b* knockout is lethal late in gestation, the role of GSK3β has been studied in *Gsk3b^+/-^* heterozygous mice, which are viable and morphologically normal. *Gsk3b^+/-^* mice display multiple behavioral abnormalities, including reduced exploratory activity, increased anxiety-associated behaviors, and reduced aggressive behavior (O’Brien et al., 2004; Kimura et al., 2007). *Gsk3a* null mice also exhibit decreased exploratory activity, increased sensitivity to environmental cues, and reduced aggressive behavior, but unlike *Gsk3b^+/-^* mice, inactivation of GSK3α is associated with impaired motor coordination, social motivation, and associative memory (Kaidanovich-Beilin et al., 2009). Although behavioral difference of *Gsk3a^-/-^* mice and *Gsk3b^+/-^* mice implies different actions of the GSK3 isoforms, in most cases, such approaches are insufficient to provide a formal proof for isoform-specific functions because it is unclear if the outcomes are attributed to lower total levels of GSK3 or to isoform-specific effects.

By using siRNAs that specifically knock down the target isoform, this study supports the notion that GSK3α and GSK3β play distinct roles in the developing brain. In particular, GSK3α and GSK3β differentially control the genesis of IPCs and further differentiation into postmitotic neurons. Although knocking down of either GSK3α or GSK3β similarly enhanced BrdU^+^-S phase cells, depletion of GSK3β but not GSK3α prevented the conversion of NPCs to IPCs. Further differentiation into Cux1^+^ neurons was also suppressed specifically by knocking down GSK3β but not GSK3α. These results suggest that GSK3β-depleted cells were arrested at the radial progenitor stage, while GSK3α-depleted cells were still able to differentiate into IPCs and Cux1^+^ postmitotic neurons. When analyzed at E18.5, fewer cells depleted with GSK3α reached the CP compared to control, suggesting that although GSK3α-depleted cells had acquired the proper laminar markers, they were unable to migrate properly. These results suggest that GSK3α and GSK3β play overlapping but distinct roles in the developing neocortex. For examination of isoform-specific roles, we confirmed that the relative degree of knockdown induced by siGSK3α and siGSK3β was similar in cell lines, primary neurons, and developing brains, and we carried out all of the experiments side-by-side to ensure identical experimental conditions. However, quantification of protein levels from immunoblots and immunostaining data is an approximation at best, and such methods are not suitable to provide truly quantitative values for unambiguously measuring the relative expression of target proteins. Although we cannot entirely exclude the possibility that a subtle difference in knockdown efficiency contributed to a difference in the observed phenotype, we favor the hypothesis that the distinct outcomes induced by knocking down one of the two isoforms are not simply due to a dosage effect. Several lines of evidence support this notion. First, as mentioned above, knocking down efficiency of siGSK3α and siGSK3β was quite similar (see **Figure 1**). Second, knocking down GSK3α and β regulated the generation of Tbr2^+^ cells in an opposite direction, rather than producing a similar trend with differing extents (see **Figure 4**). Third, depletion of only GSK3β but not a stabilized β-catenin (see **Figure 8**).

A previous study showed that complete removal of GSK3β from the progenitor cells of the developing brain in a *GSK3a* null background resulted in massive hyperproliferation of radial progenitors, inhibition of neurogenesis, and accumulation of GSK3 substrates (c-Myc and β-catenin), but neither *Gsk3a* null nor *Gsk3b^loxP/loxP^*; *nestin-cre* mice caused major brain developmental malformations (Kim et al., 2009). Such results from the double knockout mice are entirely consistent with our findings from siRNA experiments where we knocked down both GSK3α and GSK3β. However, in the present study, we show that knocking down GSK3β alone is sufficient to recapitulate almost all of the defects induced by double knockdown. At this point, the reason for the discrepancy is unclear. Although we used siRNAs designed to prevent off-target effects (ON-TARGETplus SMARTpool siRNAs), we cannot entirely rule out off-target effects of the knockdown reagents. However, many other studies have also revealed profound differences between the phenotypes caused by genetic mutations (knockouts) and those caused by gene knockdowns and suggested that the disparities can be attributed to the activation of genetic compensation in the former but not the latter (De Souza et al., 2006; Rossi et al., 2015). In single knockouts, which are devoid of either GSK3α or GSK3β function throughout development, compensatory mechanisms can be induced that are sufficient to buffer against deleterious phenotypes in the developing cortex. However, in animals where siRNAs against a single isoform are transfected into only a small population of cells, we speculate that GSK3α or GSK3β function is inhibited before the putative compensatory network can be fully induced.

If GSK3α and GSK3β play different roles, an important question remaining is how the two genes differentially regulate cortical development and brain function. It is well known that isoforms can differ in subcellular localization, temporal and spatial expression, regulatory mechanisms, biological activity, or any combination thereof. It is possible that GSK3α and GSK3β become activated or inactivated by different upstream signaling pathways at distinct layers of the developing cortex, or they regulate distinct subsets of substrates or downstream molecules. In the developing nervous system, the upstream cues and the downstream mediators are dynamically changing, all of which can affect the complex interplay.

Accumulating evidence supports the association between GSK3 with a broad range of neurological disorders (Klein and Melton, 1996; Emamian et al., 2004; Mao et al., 2009; Li and Jope, 2010; Valvezan and Klein, 2011). It will be interesting and important to determine if a particular isoform of GSK3 is more tightly linked to a subset of such diseases. As more is learned about differential regulations and actions of GSK3α and GSK3β, it may be possible to devise more specific therapeutic interventions for a number of neurological disorders associated with GSK3 signaling.

## Author Contributions

Y-xM, X-lW, J-qC, BL, E-MH, and S designed the experiment. Y-xM performed the experiments and analyzed the data. Y-xM, E-MH, and S co-wrote the paper with all authors’ input.

## Conflict of Interest Statement

The authors declare that the research was conducted in the absence of any commercial or financial relationships that could be construed as a potential conflict of interest.

## Abbreviations

BrdU: 5-bromo2-deoxyuridine
CAD: cath.-a-differentiated
Cux1: cut-like homeobox 1
CP: cortical plate
FBS: fetal bovine serum
GAPDH: glyceraldehyde-3-phosphate dehydrogenase
GFP: green fluorescent protein
GSK3: glycogen synthase kinases 3
IPCs: intermediate progenitor cells
IZ: intermediate zone
NPCs: neural progenitor cells
RGCs: radial glial cells
SVZ: subventricular zone
Tbr2: T-brain gene-2
VZ: ventricular zone
