# Supplementary figures and images for: Differential Roles of Glycogen Synthase Kinase 3 Subtypes Alpha and Beta in Cortical Development
Source: Front Mol Neurosci. 2017 Nov 28;10:391. doi: 10.3389/fnmol.2017.00391 (PMC5712306; doi:10.3389/fnmol.2017.00391)

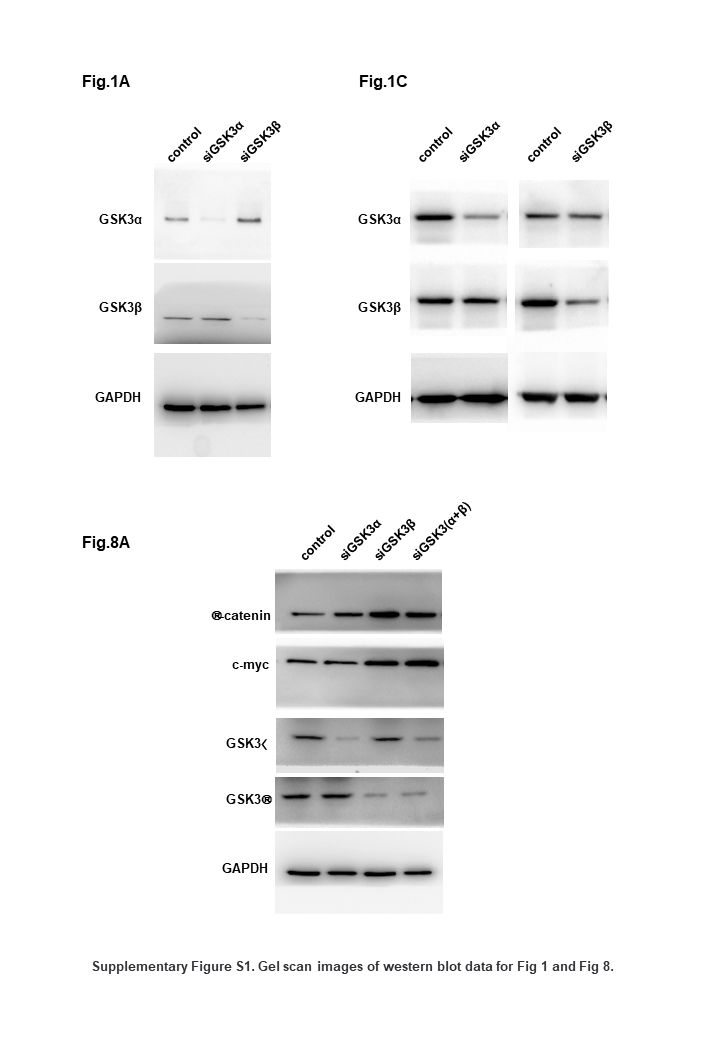

Supplement: Supplementary file 1 [file Image_1.JPEG]

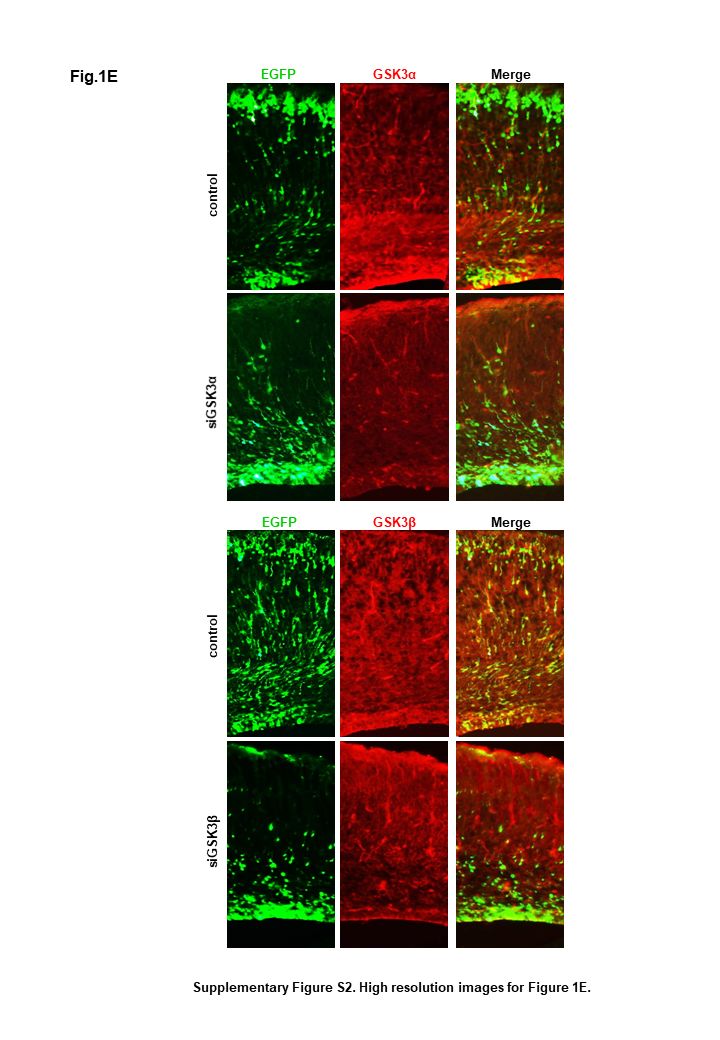

Supplement: Supplementary file 2 [file Image_2.JPEG]

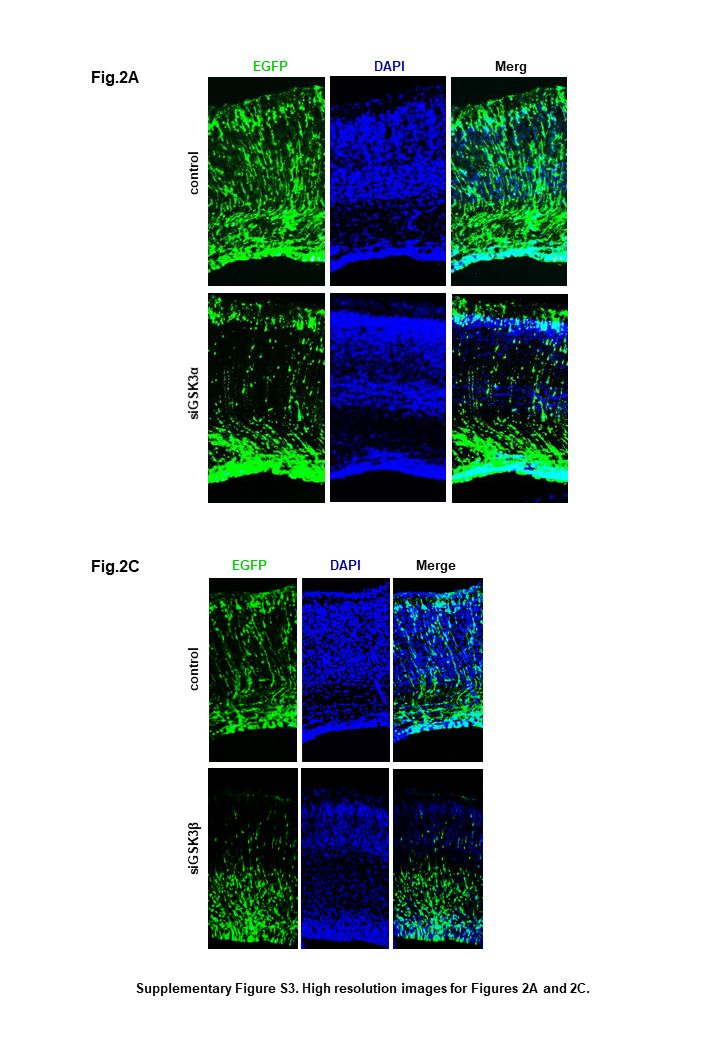

Supplement: Supplementary file 3 [file Image_3.JPEG]

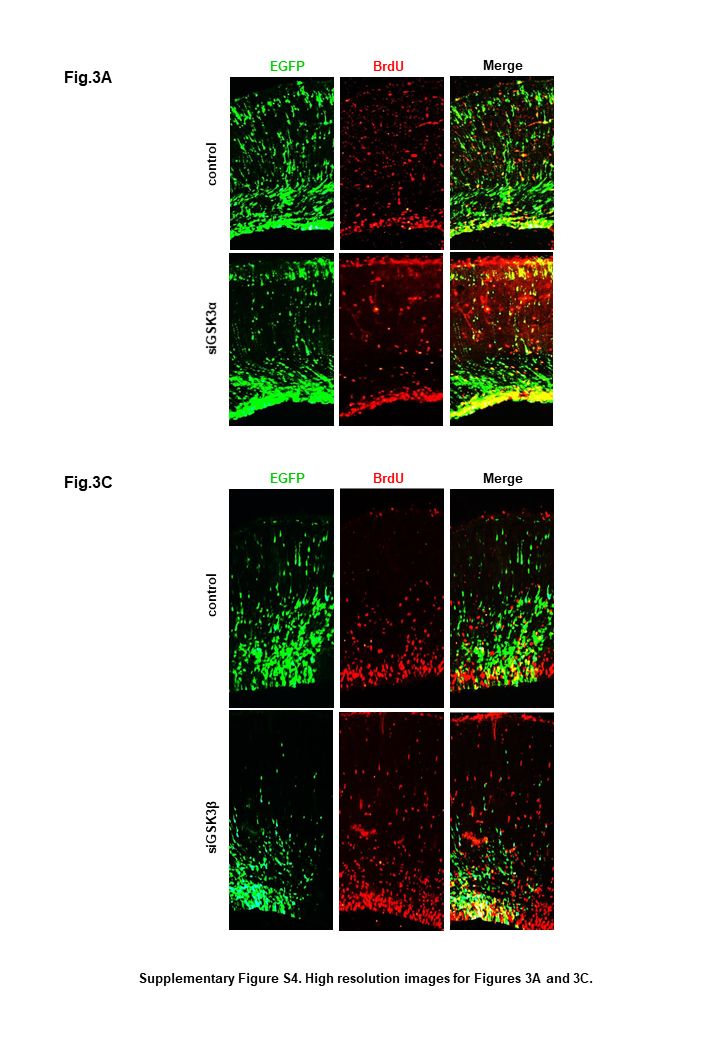

Supplement: Supplementary file 4 [file Image_4.JPEG]

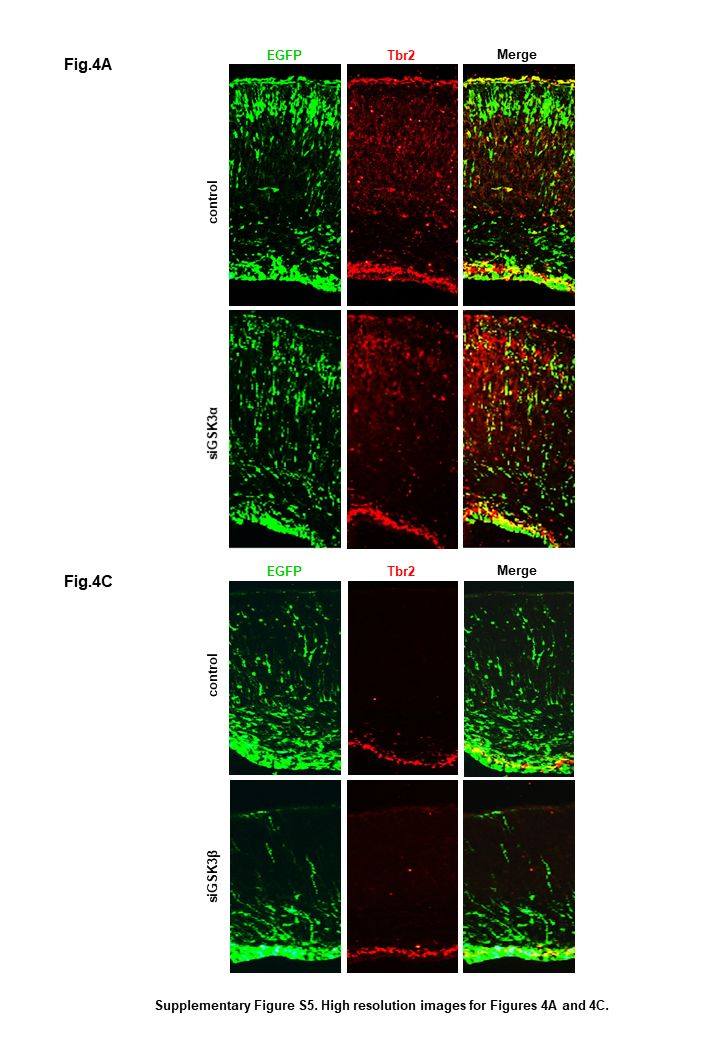

Supplement: Supplementary file 5 [file Image_5.JPEG]

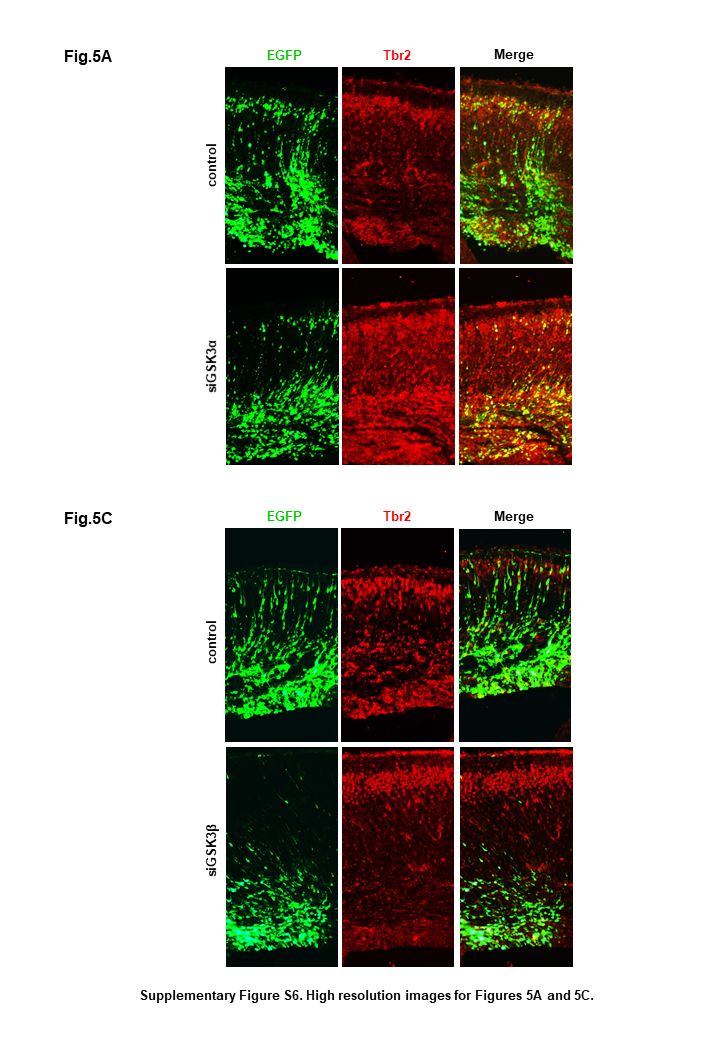

Supplement: Supplementary file 6 [file Image_6.JPEG]

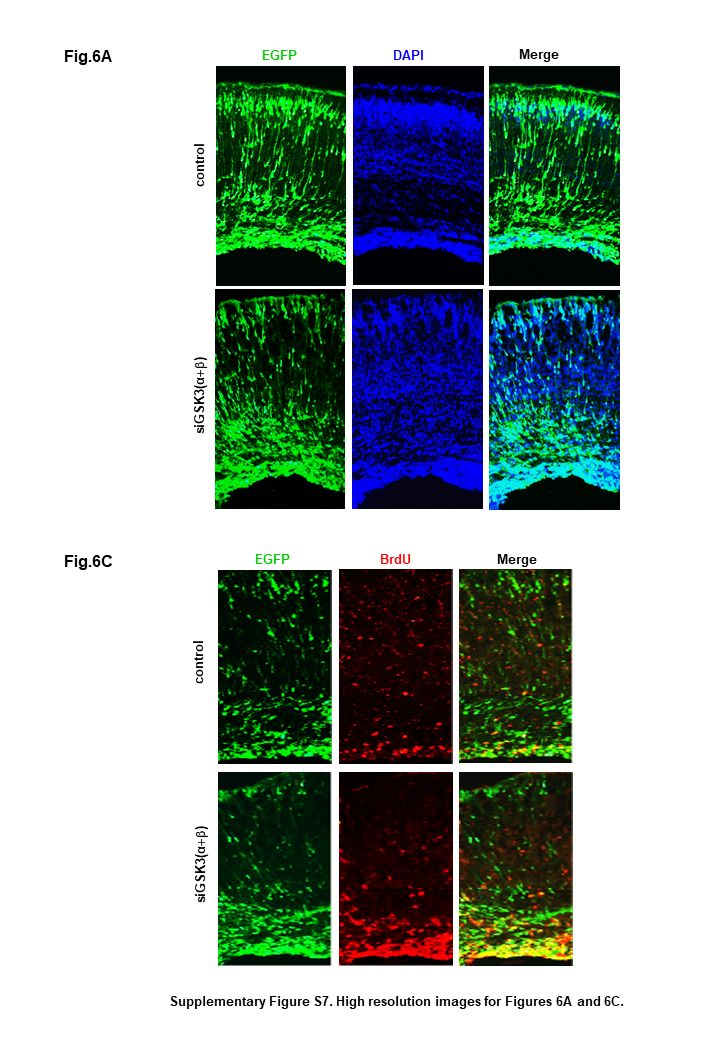

Supplement: Supplementary file 7 [file Image_7.JPEG]

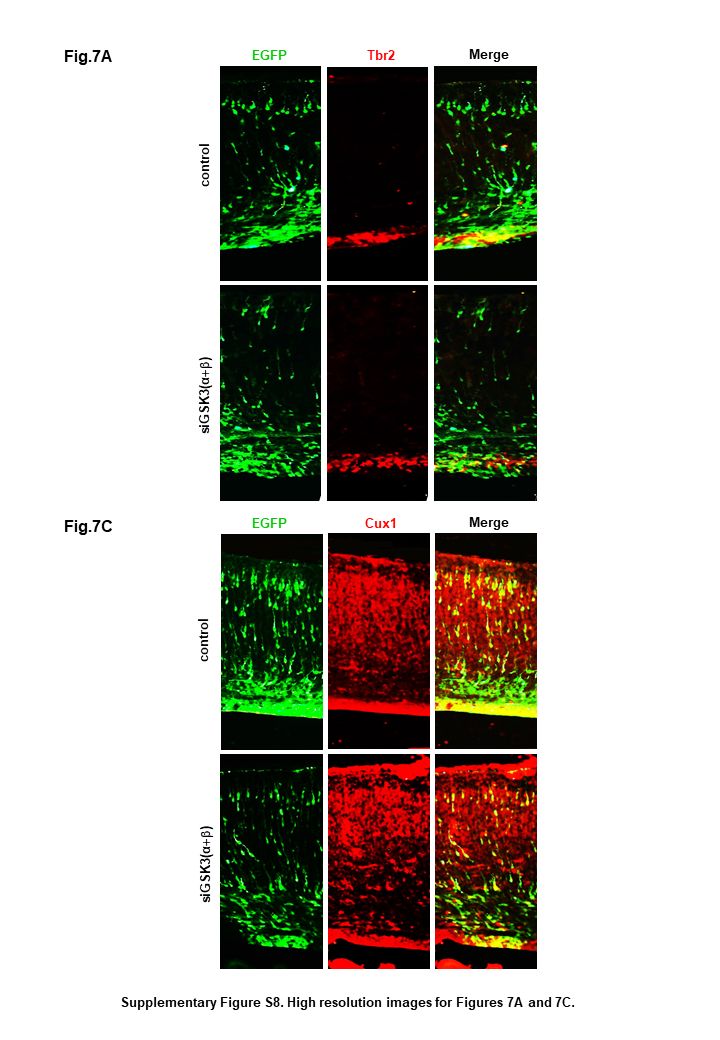

Supplement: Supplementary file 8 [file Image_8.JPEG]

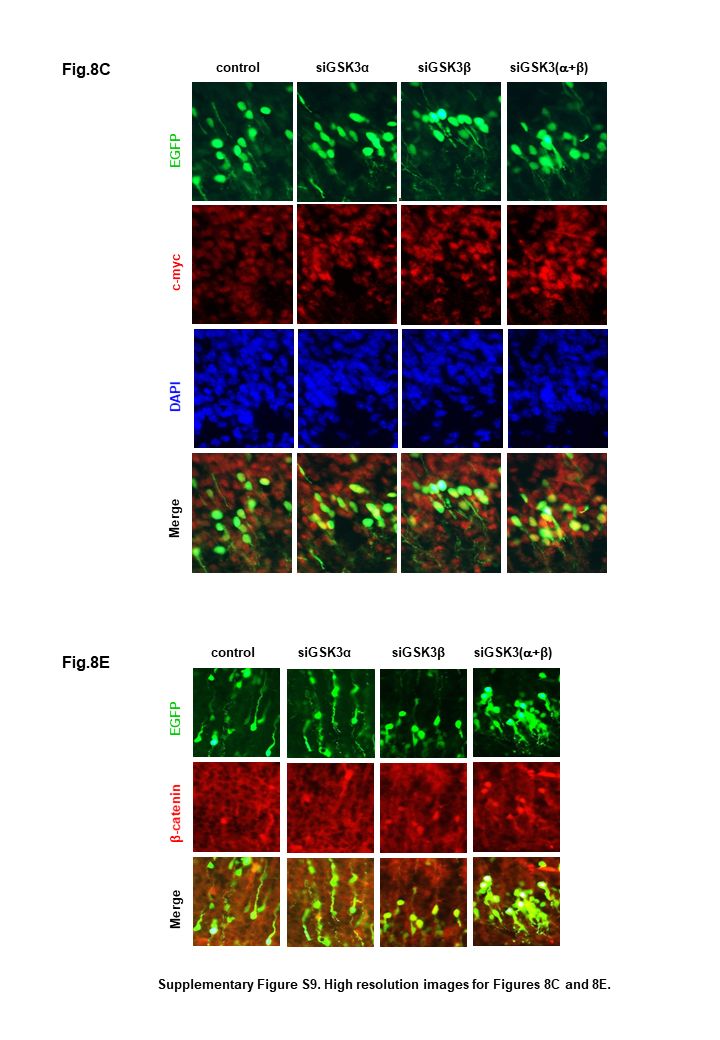

Supplement: Supplementary file 9 [file Image_9.JPEG]
